# Predictors of prenatal iron folic acid supplement utilization in Wolaita, South Ethiopia: a community based cross-sectional study (quantitative and qualitative approach)

**DOI:** 10.1186/s12884-020-02883-2

**Published:** 2020-04-25

**Authors:** Ermias Wabeto Wana

**Affiliations:** Offa District Health Office, Wolaiata Zone, Soddo, South Ethiopia Ethiopia

**Keywords:** Prenatal, Iron supplementation, Pregnant, Utilization, Ethiopia, ANC

## Abstract

**Background:**

Effectiveness of prenatal iron supplementation program depends on utilization of the supplement by pregnant women. Nevertheless, in Ethiopia, regardless of increasing efforts, lower proportion of pregnant women use the supplementation for recommended 3 months and above but, the reason is not clear. This study aimed to identify the level and factors associated with utilization of prenatal iron supplementation.

**Methods:**

A community based cross-sectional quantitative study complemented with qualitative component was carried out in January, 2016. A total of 411 pregnant women who gave birth 6 months preceding data collection were selected using multi-stage cluster sampling technique. Qualitative data were collected by conducting four focus group discussions (FGDs) of local pregnant mothers and interviewing district maternal and child health (MCH) focal, 3MCH heads of health centers and four health extension workers. Factors associated with appropriate utilization of supplementation were assessed using multivariate logistic regression. The outputs of analyses were presented using Adjusted Odds Ratio (AOR) with 95% confidence intervals (CI).

**Result:**

From the study participants, 11.5% (95% CI = 9.9–13.1%) took the supplement for the recommended duration of 3 months or above. Pregnant women who could not read and write had 77% reduced odds of using iron supplementation than their counterparts (AOR = 0.23(95% CI: 0.07–0.75)). As compared to women who had four or more antenatal care (ANC), women with 2 and 3 ANC visits had 78% (AOR = 0.22(0.07–0.63)) and 66% (AOR = 0.34(0.14–0.81)) reduced odds of using the supplementation, respectively. As compared to women who were aware of benefits of taking the supplement for maternal and child health, the counterparts had 90% (AOR = 0.10 (0.10–0.63)) reduced odds of using the supplement. Women who were not knowledgeable of anemia had 85% (AOR = 0.15(0.04–0.62) reduced odds of using the supplement than those who were knowledgeable. The qualitative study indicated that there was no problem in the supply and logistic system of iron supplement and leading reasons for not taking the supplement were late initiation of Antenatal Care, lack of awareness and occurrence of side effects; unpleasant taste, nausea, vomiting.

**Conclusion:**

In the study area utilization of prenatal iron supplementation is very low. Improving maternal education, ensuring early and frequent ANC, educating pregnant women about the benefits of service and ensuring comprehensive knowledge of anemia expected to improve the utilization of prenatal iron supplementation.

## Background

Anemia affects more than 2 billion people of the world and majority of the burden is attributed to iron deficiency (ID) [1]. ID represents spectrum that ranges from asymptomatic depletion of store to apparent iron deficiency anemia (IDA) [1]. The hemoglobin concentration less than 11 g/dl in pregnancy is termed as anemia [2]. About 42.0% of pregnant women worldwide are anemic and half of this anemia is attributed to ID [3]. ID can result from inadequate intakes, absorption and increased physiologic iron requirement during pregnancy because it is needed for fetus and placenta development, store in fetus for the first 6 month of life after delivery and expansion of maternal blood volume [4]. Pregnant women are at special risk of Iron deficiency or Iron deficiency anemia due to higher iron requirement and their need is cannot be satisfied by dietary intake alone; thus, iron supplementation has become an essential component of maternal health programs [5]. The Ethiopian pregnant women are also at higher risk of anemia as compared to non-pregnant women; in-depth analysis of Ethiopian demographic and health surveys (EDHS) of 2000 to 2011 showed that, 33.1% of pregnant women were anemic as compared to 18.3% of non-pregnant counterparts 5 years preceding 2005 EDHS and 29.9% of pregnant women were anemic as compared to 10.8% of non-pregnant women 5 years prior to 2011 EDHS [6].

Ethiopian national nutrition strategy (NNS) targeted to increase proportion of pregnant women who take iron supplementation for 3 month or more to 50.0% by 2015 [7]. However, smaller proportion of pregnant mothers take the supplement for 90 days or more during their pregnancy; less than 1% took for 3 month or more [8] and as study carried out in four major regions of Ethiopia, only 3.5% took for the recommended duration [9]. The study carried out in Mecha district showed that about 20.4% of pregnant women took for 90 plus days during their recent pregnancy [10].

There is no conclusive and consistent evidence in Ethiopia. Moreover, there is no well documented information on the level and factors associated with utilization of iron supplementation. Thus, purpose of the current study was to determine the proportion and factors associated with utilization of prenatal iron supplementation in wolaita zone, south Ethiopia.

## Methods

### Study design and period

Community based cross-sectional study using quantitative study design complemented with qualitative approach was conducted via home to home visit in January 2016.

### Study setting

The study was carried out in Offa woreda which is found in Wolaita zone 362 Km to the South of Addis Ababa and 185 Km to the South West of Hawassa (capital city of SNNPR). According to 2007 Central Statistical Agency (CSA) report, the Woreda has estimated total population of 135,527; with an estimated male to female ratio of 1to1 and 4878 pregnant mothers. There were 4 health centers and 27 health posts that serve the population with potential health services coverage of 73.8%. According to health management information system (HMIS) report, about 97.6 and 81.5% of pregnant women got first and fourth ANC services, respectively and the annual health professional assisted delivery coverage was 59% in 2015 [11].

### Source and study population

The source and study populations of the study were women who gave birth 6 months preceding data collection and living in entire woreda and randomly selected kebeles (small administrative division of the Woreda) respectively.

### Sample size calculation

The sample size for quantitative study was calculated by using EPI info version7 for windows using single population proportion formula using the following assumptions; The expected proportion of utilization for 91 or more days 20.4% [10], 95% significance level, 5% margin of error, 1.5 design effect due to 2 stage cluster and 10% expected non-response rate gave sample size of 411.

### Sampling technique

Randomly 10 kebeles were selected by simple random sampling (SRS) technique. Total number of sample required from each randomly selected kebele was proportionally allocated. The participants were selected by SRS from list of women who gave birth in 6 months preceding data collection which was established prior to data collection by conducting listing through mobilization of health development army (HDA). Eight to ten pregnant mothers for each FGD were purposively selected from list of antenatal care attending women.

### Data collection

#### Quantitative data collection

A structured questionnaire was prepared in English (Additional file 1) translated to the Amharic language. Another translator then translated the Amharic version back into English to check for consistency of meaning. The study variables were adopted from the relevant literature.

Data were collected by trained diploma nurses with close supervision by supervisor and the principal investigator. The interviewer administered Amharic version questionnaire was administered to randomly selected women to collect quantitative data via home to home visit.

Data collectors were given one-day training on the study objectives, method of data collection, and the tools for data collection. The supervisor was senior public health expert and has conducted onsite supervision on the quality of data collection in the field and submitted completed questionnaires to the principal investigator on daily basis.

#### Qualitative data collection

Qualitative data were collected via recording in-depth interview of district maternal and child health (MCH) focal person, MCH department heads of health centers and Health extension workers (HEW) and FGD among pregnant women. Interviews and FGDs on each topic facilitated using semi-structured guideline that included some questions from quantitative part that need further exploring. In-depth interview was undertaken in respective working places of the interviewees and ultimately eight interviews among district MCH focal person (1), health center MCH heads (3) and HEWs (4) were carried out. Participants for FGD were selected purposively from pregnant women who attended ANC at once, twice, thrice and four times during study period and four FGDs were undertaken. All the discussants had been informed on the purpose of the discussion, why we take the record, coached to be in line with discussion topic and encouraged to freely participate in discussion. Beside recording, note had been taken on some pertinent information that cannot be recorded like facial expression and gesture while discussion was proceeding. Information maturation (the point where no new thought was coming out) was considered during both interviews and FGDs.

### Operational definition

- Knowledge of anemia: women who have heard of anemia and know at least one symptom, cause, prevention method and consequence of anemia.
- Late pregnancy: time period after second trimester of pregnancy.
- Utilization of iron supplementation: Proportion of pregnant women who took iron supplement for 3 month or more.

### Data management and analysis

The raw data were checked for completeness and consistency and had been given individual unique identification number and then entered into Statistical Package for Social Science (SPSS) version 20. The wealth index (indicator of living standard of household) was constructed through principal component analysis (PCA). Attitude towards utilization of iron supplementation and anemia was assessed by using health belief model (HBM) constructs [12]; these were perceived susceptibility to anemia, perceived severity of anemia that occur during pregnancy, perceived benefits and barriers of taking iron supplementation. Level of perception was assessed by using Lickert scale (very disagree, disagree, neutral, agree and very agree). Descriptive statistics was used to describe the study variables and logistic regression was undertaken to identify the factors associated with utilization of prenatal iron supplementation. Multi-colinearity was checked to test the interrelation between predictor variables and chi-square test was carried out to check cells adequacy. Findings of descriptive analysis were given by percentage, mean or median and the logistic regression outputs of SPSS were given by crude odds ratio (COR) and AOR with their 95% CI. Socio-demographic Variables, health service access and utilization variables, personal/behavioral variables were used as predictive variables in multi-variate analyses. The variables in bi-variate analyses were entered into multivariable analyses to identify the statistical significance among dependant and explanatory variables. *P*-value of less than 0.05 was taken as the statistical significance level.

The information recorded during qualitative data collection was first transcribed into hard copy document. Both the transcription and notes taken in the field had been read thoroughly and related ideas were summarized together to develop themes. Themes were arranged in their logical order and finally results had been narrated manually making the use of themes and quoting ideas that were strongly expressed during interview and FGD.

### Quality assurance

Before collection of the actual data, 5% of total sample size was pretested and necessary corrections on questionnaires were made accordingly. Translating of questionnaire from English to Amharic and back to English to check consistency was made. At the end of each data collection day, data were checked for completeness and consistency and discussion with the research assistants was undertaken. Information saturation and homogeneity of FGD participants have got concern.

## Results

### Socio-demographic characteristics of the respondents

Four hundred one women who gave birth 6 months preceding the data collection responded the questionnaire successfully and the response rate was 97.6%. Mean age (±SD) of the respondents was 27.4 (±4.4) years. The median (IQR) number of children ever born to a woman was 3 (2) and about 15.5, 71.8 and 12.7% of respondents gave birth to one, two to four and five or more children, respectively. The majority of respondents were in the age range between 25 and 34 years (Table 1).

**Table 1:** Socio-demographic characteristics of the respondents, Wolaita, South Ethiopia, January 2016

Socio-demographic characteristics (*n* = 401)	Frequency	Percent
Age in years		
24 or below	95	23.7
25–34	279	69.6
35 or above	27	6.7
Religion of the respondents		
Protestant	300	74.8
Orthodox	94	23.5
Others	7	1.7
Marital status		
Married	389	97.0
Others	12	3.0
Maternal education status		
Illiterate	190	47.4
Primary (grade 1–8)	152	38.0
Secondary (grade 9–12)	49	12.2
Higher education (grade above 12)	10	2.4
Occupational status		
Housewife	235	58.6
Merchant/business	84	20.9
Farmer	65	16.2
Daily laborer	10	2.5
Salaried employee	7	1.7
Spousal educational status		
Illiterate	149	37.2
Primary (grade1–8)	135	33.6
Secondary school (grade 9–12)	99	24.7
Higher education (grade above 12)	18	4.5

### Antenatal care service utilization

More than three fourth (75.6%) of participants attended ANC at least once during the recent pregnancy and. Mean (±SD) gestational age at first ANC visit among respondents who had at least one ANC was 5.1 (±1.7) months. Forty four percent received ANC from the health post and 17% had the recommended four or more ANC visit during the index pregnancy (Table 2).

**Table 2:** Antenatal care utilization, place of visit, GA at first ANC and frequency of ANC visit among women who gave birth in the last 6 months, Wolaita, South Ethiopia, January 2016

Antenatal care Service	Frequency	Percent
ANC follow up (*n* = 401)		
Yes	303	75.6
No	98	24.4
Place of visit (*n* = 303)		
Health post	177	58.4
Health center	124	40.9
Hospital	2	0.7
GA at first visit (*n* = 303)		
1–3 month	50	16.5
4–6 Month	185	61.1
≥ 7 month	68	22.4
ANC frequency (*n* = 303)		
Once	27	8.9
Twice	88	29.0
Thrice	120	39.6
Four or more	68	22.4

Respondents who had ANC visit reflected on the quality of ANC services. They perceived service quality as poor 4.5%, neutral 7%, good 6.7% and very good 27.4%. More than half (51.4%) of respondents perceived that they can face life threatening conditions during pregnancy.

### Knowledge and access to information about anemia

Out of the entire respondents, 62.3% women had ever heard of anemia and they mentioned symptoms of anemia; tiredness 50.1%, pale appearance 35.4% and loss of appetite 32.9%. Concerning the causes of anemia during pregnancy, 59.9% mentioned failure to take iron supplementation, 42.6% identified inadequate dietary iron intake as a cause. Other causes reported were blood loss 14% and parasitic infection 13.7%. Most frequently mentioned anemia prevention methods were taking iron supplementation 59.4% and proper maternal nutrition 43.1% and other prevention mechanism mentioned was preventing parasitic infection 13.7%.

Regarding the respondents knowledge towards consequences of anemia, 45.1% reported that anemia increases likelihood of maternal death, 41.1% anemic women become seriously ill and 9 and 4.7% mentioned that anemia can result in fetal death and LBW, respectively.

Knowledge of anemia among respondents was assessed based on whether they heard of anemia or not and knowledge of symptoms, causes, prevention methods and consequences of anemia they had. Woman was categorized to be knowledgeable of anemia if she heard of anemia and knows at least one symptom, cause, prevention and consequences. Regarding this, 50.6% women were knowledgeable of anemia. From those heard of anemia, 96.8% could mention at least one symptom of anemia (Table 3).

**Table 3:** knowledge of anemia among women who gave birth in the last 6 months, Wolaita, South Ethiopia, January 2016

Knowledge characteristics	Frequency	Percentage
Ever heard of anemia (*n* = 401)	250	62.3
Of those heard of anemia, knows at least one symptom (*n* = 250)	242	96.8
Of those heard of anemia, knows at least one cause (*n* = 250)	223	89.2
Of those heard of anemia, knows at least one prevention method (*n* = 250)	243	97.2
Of those heard of anemia, knows at least one consequences (*n* = 250)	223	89.2

### Knowledge about iron supplementation

Among the respondents, 80.5% had ever heard of iron supplementation and 72.1% have been informed about it during their recent pregnancy. The sources of information were HEWs 38.2%, health professionals 34.4%, mass media 27.9% and HDA members 4.7%. About 27.9 and 4.5% of respondents have received home to home visit care including information about iron supplementation from HEWs and HDAs, respectively.

From the respondents who had at least one ANC visit, 91.1% had been informed about the significance of iron supplementation. Type of information they get were iron supplementation prevents anemia 74.9%, all pregnant women need to take it regardless of their anemia status 34.0%, taking iron supplementation can cause side effects 19.1% and how to manage side effects if happened 2.6%.

### Attitude of the respondents towards iron supplementation

Attitude of the study participants regarding utilization of prenatal iron supplementation and anemia was assessed using health belief model (HBM) constructs [12]. On this regard, respondents perceived that pregnant woman who does not take iron supplementation is at risk of developing anemia 64.3%, taking iron supplementation during pregnancy improves maternal and child health 73.6%, anemia that occur during pregnancy can seriously affect maternal and child health 93.3%. Barriers of taking iron supplementation was assessed based on woman’s perception on side effects, family rejection of taking iron supplementation and perceived negative effects on fetus. About 56.2% women worried that taking the supplement results in undesirable effects, 18.5% thought negative effects up on the fetus and 5.0% felt that family rejects taking the supplement.

### Iron supplement utilization

Out of the entire respondents, 70.1% took iron supplement at least once during their recent pregnancy. The median number of days the respondents had taken iron supplementation was 50 (IQR = 45). The length of days which women have taken iron during their pregnancy period ranges from 4 to 140 days. From all the study participants, only 11.5% (95% CI = 9.9–13.1%) took the supplementation for the recommended duration of 3 months or above [13, 14].

Among those who took iron supplementation at least once, nearly three fourth (76.5%) have been pre-informed about the total number of days iron supplements have to be taken. Of the respondents those have been taking the supplement, 68.3 and 11.2% got information on benefits and possible side effects, respectively. Amongst those who took a supplementation at least once, more than three fourth (86.8%) reported that they have been advised to take the supplementation on daily bases.

Among women who had taken iron supplement at least once during the recent pregnancy, 13.9, 60.1 and 26.6% started taking iron supplementation within first, second and third trimesters respectively. Reasons for starting late in the pregnancy (after second trimester) were delayed initiation of ANC visit 43.2%, having no information about iron supplementation 36.5%, no specified reasons16.2% and others 4.1%.

Regarding the source of iron supplements, 34.5% respondents took from health post, 34% from health center and 0.7% from hospital. About 8.2% reported that they encountered a moment when they got no or inadequate iron supplement during their visit to health facility. About 86.5% of respondents who have been prescribed iron supplementation during their recent pregnancy period were reminded about taking iron during their consecutive ANC visits. Utilization of recommended dose of IFA is determined by mother’s adherence towards recommendation by the health worker. Among the respondents who had taken iron supplementation at least once, 55.2% reported that they have been taking the supplementation on daily bases while the remaining 44.8% reported otherwise. Frequently mentioned reasons for missing the supplement were forgetfulness (46.4%), side effects (26.4%), finding it difficult to take the supplement daily (16.8%), with no specific reason (8.0%) and others (2.4%). Of the respondents who started taking the supplement, 83.6% stopped taking iron supplementation at some point before reaching the recommended 91 days plus. The main reasons for discontinuation were side effects (39.8%), birth of the baby (25.4%), fear of taking recommended number of tablets during pregnancy (22.9%), fear of fetal weight gain (4.7%), fear of other health problems (5.5%) and others (1.7%). Amongst the women who took the supplement 60.1% complained of side effects due to taking the supplementation; unpleasant taste (50.9%), nausea (47.3%), vomiting (29.6%), gastric irritation (24.3%), diarrhea (9.5%) and constipation (5.9%).

From the entire respondents, 29.9% did not take iron folic acid supplementation during the index pregnancy. Reasons for not taking at all were; had no information regarding importance of iron (12.7%), fear of side effects (4.2%), don’t know where to get the supplement (4.5%), dislike the taste (2.7%), due to religious reasons (2.2%), unavailability of iron tablet in the health facility during time of the visit (2.1%) and assume getting enough iron from food (1.5%).

### Variables/factors associated with utilization of prenatal iron supplementation

Maternal age, birth order, maternal education, spousal education and household income, two-way distances from the nearest health facility, health service utilization, perception on taking iron and knowledge related factors were considered as variables that affect utilization of prenatal iron supplementation. Maternal education status, spousal education status, birth order, household economic status, time taken for two way trip to the nearest health facility, ANC frequency, maternal knowledge towards anemia, perceived susceptibility to anemia and perceived benefit of taking the supplement were covariates included in adjusted analysis. Educational status of women, family economic status, Number of ANC visits, maternal knowledge on anemia and mother’s perceived benefit of taking the supplement were significantly associated with utilization of prenatal iron supplementation; odds of using prenatal iron supplementation among illiterate women was 77% reduced than their counterparts (AOR = 0.23(95% CI = 0.07–0.75)). As compared to women in the richest wealth tertile, those in poorest tertile had 4.43 times increased odds of utilizing the supplementation (AOR = 4.43(95% CI = 1.74–11.28)). As compared to women who had four or more antenatal care (ANC), those who conducted 2 and 3 ANC visits had 78% (AOR = 0.22(0.07–0.63)) and 66% (AOR = 0.34(0.14–0.81)) reduced odds of using the supplementation, respectively. Women who lack comprehensive knowledge of anemia had 85% reduced odds of utilization than their counterparts (AOR = 0.15 (0.04–0.62)). As compared to women who perceived that taking iron supplements improves maternal and child health, the counterparts had 90% reduced odds of using the supplementation (AOR = 0.10(0.01–0.63) (Table 4).

**Table 4:** Factors associated with utilization of prenatal iron supplementation Wolaita, south Ethiopia, January 2016

Variables	Iron supplementation Used (*n* = 46)	Iron supplementation not used (*n* = 355)	COR (95% CI)	AOR (95% CI)	*P*-Value
Age in years					
15–24	9	86	1	–	–
25–34	33	246	1.28 (0.58–2.78)	2.17 (0.76–6.23)	0.14
35 or above	4	23	1.66 (0.56–5.88)	5.31 (1.08–25.96)	0.04
Maternal education					
Illiterate	6	184	0.14 (0.06–0.34)	0.18 (0.05–0.59)**	0.005
Literate	40	171	1	1	1
Birth order					
Primigravida	9	53	1.38 (0.63–3.03)	1.2 (0.38–3.76)	0.74
Multigravida	37	302	1	1	1
Spousal education					
Illiterate	12	139	0.33 (0.15–0.72)	1.90 (0.56–6.42)	0.30
Literate	34	216	1	1	
Wealth index					
Poor	32	129	4.68 (2.08–10.52)	4.58 (1.75–11.90)**	0.002
Middle	6	75	1.51 (0.51–4.51)	2.35 (0.65–8.42)	0.19
Rich	8	151	1	1	
Double trip distance from nearest health facility					
Up to 30 min	34	188	1	1	
31–60 min	10	137	0.40 (0.19–0.85)	0.76 (0.31–1.91)	0.56
More than 60 min	2	30	0.37 (0.08–1.59)	0.21 (0.0.04–1.20)	0.08
ANC frequency					
0	2	96	0.04 (0.01–0.19)	0.25 (0.04–1.46)	0.12
1	2	25	0.17 (0.04–0.77)	0.28 (0.04–1.20)	0.20
2	7	81	0.18 (0.07–0.45)	0.24 (0.08–0.69)**	0.009
3	13	107	0.25 (12–0.55)	0.32 (0.13–0.79)**	0.014
4 or more	22	46	1	1	
Knowledge of anemia					
Knowledgeable	41	162	1	1	
Not knowledgeable	5	193	0.10 (0.04–0.27)	0.15 (0.03–0.52)**	0.005
Perceived risk of anemia					
Yes	36	222	1	1	
No	10	133	0.46 (0.22–0.97)	0.96 (0.32–2.91)	0.94
Perceived benefit of taking iron supplementation					
Yes	45	250	1	1	
No	1	105	0.05 (0.01–0.39)	0.10 (0.01–0.63)**	0.02
Perceived barriers of taking iron supplementation					
Yes	28	219	1	1	
No	18	136	1.04 (0.55–1.94)	2.88 (1.10–7.54)	0.03

Utilization of the supplementation is using/taking iron folic acid supplement for 91 days or more

**explanatory variables significantly associated with dependent variable in multivariable model (*p* < 0.05)

### Qualitative results

#### ANC service utilization

The interviewees (HEWs, midwifery nurses and program coordinating person at health Office) mentioned that ANC service components utilization is improving in the Woreda over time. The main underlining reason is successful promotion activity through HEWs and HDAs. HDA members are involved in identification and listing of pregnant women in their respective catchment areas so that pregnant women can book for the service. Conducting home to home visit by HEW and HDAs and encouraging pregnant women to undertake visit also facilitates ANC service utilization. All discussants agreed that ANC follow-up is necessary to every pregnant woman regardless of her health status as risk factors are unpredictable.

Regarding time of initiating ANC visit, majority of the discussants claimed that they started follow-up late after 5 month of pregnancy. Health professionals and HEWs also explained that majority of the pregnant women in their respective catchment health facilities start ANC visit late. The reported reasons for doing so were lack of awareness, late recognition of their pregnancy by the women themselves, late identification of pregnancy by health care providers and lack of access to health institutions. When women start ANC visit late, the duration of taking the supplement is shortened because most of them stop taking after delivery of the baby.

#### IFA utilization and related information

Most of the health care providers stated that they were providing iron folic acid supplement to pregnant women. Regarding information being provided to the beneficiaries during iron provision, benefit of taking iron and how many tablets to take once were frequently mentioned.

A midwifery nurse in health center said that *“we also advice the beneficiaries to delay taking tea/coffee after taking the supplementation”.* The information had been provided focused on benefit that can be obtained from taking iron; no discussant had information on side effects that can occur due to taking the supplement, how to manage them and total tablets to be taken.

Sickness in general and pregnancy itself were most frequently mentioned cause of extreme tiredness and fatigue. Few of the FGD participants mentioned that anemia can cause extreme tiredness and fatigue.

*“Anemia is a disease that results in extreme tiredness and fatigue; even it is not possible to anemic women to stand up and sit down” (23 years old pregnant woman who completed grade 10 at school).* Anemia can increase risk of maternal death, can damage the fetus and result in severe illness among mother were frequently mentioned consequences among FGD members. Discussants had fairly good knowledge level on anemia, its consequences and many of them agreed the possibility of preventing anemia through taking iron supplementation.

Interviewed health workers agreed that all pregnant women are in need of iron supplementation and mentioned that majority of pregnant women in their catchment were using the supplement. Similarly most of the FGD discussants knew iron tablet and why it is given for.

Majority of the FGD participants discussed as they were using iron supplementation;

*“Whatever the living standard of the pregnant women is, she is at risk of developing anemia; therefore, all pregnant women should take iron tablet” (25 years old pregnant woman who completed grade 9 at school). P*regnant discussants who were taking iron supplement told that they were happy to take the supplement.

Some member of FGD talked that they were not taking the supplementation; because, lack of awareness, perceived side effects, having no ANC visits and negligence. Others used to miss the supplement and frequently mentioned reasons for doing so were side effects like nausea, gastric irritation and forgetfulness. No cultural and religious beliefs and negative community perceptions that hinder utilization had been mentioned. The discussants mentioned that some other pregnant do not consume iron at all because they think that there is enough iron in the food they eat. HEWs and health professionals reported that they have been checking adherence of pregnant women towards iron supplementation by randomly conducting pill count during home to home visit, take self report from attendants during subsequent visits on number of tablets they have in home and compare days between previous and current visit.

HEWs and health workers reported that they did face no stock out of iron supply in the last year and they had adequate iron folic acid in their stock during interview. Health centers access iron tablet from woreda health office based on their consumption level that was managed by bin card and internal facility request and resupply form (IFRR). Health posts reported that they control their stock using bin card and access iron from their respective catchment health centers based on their need. Health workers monitor management of iron during their supportive supervision both in health posts and health centers.

The group members of respective FGD forwarded some recommendations to improve the future utilization of iron tablet among pregnant women. The majority of group members agreed on strengthening information and education provision to improve mother’s knowledge and follow-up to women to improve their adherence to iron.

*“To improve the future utilization of iron supplementation, it needs integrating activities at all level; HEW and health professionals should improve quality of counseling, empower HDAs, should early identify pregnancies, continuous follow-up and supervision from health center and district level” (Woreda MCH coordinating person).*

## Discussion

Prevention of Iron deficiency anemia (IDA) during pregnancy relies on utilization of prenatal iron supplementation. Thus, current study tried to investigate the level and factors associated with utilization of prenatal iron supplementation. The study revealed that the utilization level (taking the supplement for recommended 91 days or above) was still low and predicted by different variables including maternal education, economic status, frequency of ANC visits, knowledge of anemia and perception of women as taking iron supplement improves maternal and child health.

The proportion of women who had appropriate utilization of iron supplementation was found to be 11.5%. Even though the figure is low, it is higher than finding from recent EDHS that was less than 1% [8] and study done in four major regions of Ethiopia 3.5% [9] and this might be due to the recent strong governmental intervention to achieve the goal stated in the NNP [7], time gap during which access to different information sources is improving and they used relatively longer reference period for that reason recall might be difficult. However, level of utilization found by current study is lower than finding form study conducted in Mecha district 20.4% [10]. Probable reason may be the difference in iron tablet related information provision in study areas; about 65.6% clients were adequately advised in Mecha; whereas, only 12.2% had been advised both benefit and possible side effects related with taking the supplementation in current case.

Demographic and health surveys analysis in Indonesia [15] and study in Senegal [16] showed that utilization for 3 months plus was 34 and 58%, respectively. The figures were higher than current finding 11.5%; this may be due to difference in quality of ANC counseling; in current case, small proportion (11.2%) of women had been counseled about possible side effects related with taking iron supplement. As FGD showed, no woman had been told total days iron have to be taken and possible side effects. The other possibility might be the difference in monitoring and coordinating the coverage and iron supplementation programs in study areas. Falling within the severe public health significance level of anemia in former study areas as showed by WHO global database on anemia [17] may be contributed working hard in anemia prevention activities.

Number of studies revealed that maternal education is positively associated with utilization of prenatal iron supplementation. According to studies in Amhara region [10], Ethiopia [8], Khartoum [18], Rio de Jenerio [19] and India [20], illiterate women had reduced odds of using prenatal iron supplementation as compared to women who can at least write and read. A current study also showed the same direction of association between these variables. But, a study undertaken in the four major regions of Ethiopia [9], showed that illiterate women had increased odds of using iron supplementation during pregnancy.

It is common to report economically empowered women use increased number of iron supplementation than their counterparts. Studies undertaken in Indonesia [15], Northern Tanzania [21] and Ethiopia [8] suggested that economically better off women use increased supplementation than the poorest counterparts. But, the current study witnessed opposite direction in consistence with study carried in four major regions of Ethiopia. The probable reason for inconsistency may be economically advantaged group think that they get adequate iron from diet, may consider anemia highly affects poor parts of community as result they neglect adherence to the supplementation. On the other hand, economically disadvantaged women may doubt diet is not satisfying their iron requirement; thus, can adhere more.

As witnessed by several studies, utilization of prenatal iron supplementation increases with increasing frequency of ANC visits. Studies undertaken in Rio Grande [22], Tanzania [21], Khartoum [18], Tigray [23] and four major regions of Ethiopia [9] found that increased ANC visit enhance increased use of iron tablets. The current study showed consistent results; women who had ANC lower than four visits had reduced odds of using prenatal iron supplementation than those who had four plus ANC visits. FGD discussants also mentioned having no ANC visit reason for non-use. Intuitively, ANC follow up serve as route of providing iron supplement to pregnant women and encourage their adherence.

Though half (50.6%) of the study participants were knowledgeable of anemia; it became significantly associated with utilization of prenatal iron supplementation. Women who lack comprehensive knowledge had reduced utilization of iron supplementation. This is in consistence with studies undertaken in India [20], Kenya [24], Amhara [10] and four major regions of Ethiopia [9]. Ensuring comprehensive knowledge among women during pregnancy enhances using iron supplementation for recommended duration.

The current study revealed that perceived benefit of taking iron supplementation during pregnancy for maternal and child health had been predictor of prenatal iron supplement utilization. This goes in line with study undertaken in Senegal [16]. Study carried out in Kenya [25], also witnessed as utilization increases when women had personal need to protect oneself from anemia.

Side effects played a major role for missing, discontinuing and failure to take the supplement. It has been reason for missing (26.2%) and discontinuing the supplement (23.4%). Forgetfulness also takes a large share for missing the supplement. About 44.8% had missed (did not taking daily) the supplement and 46.8% of these were due to forgetfulness. The qualitative study also found that side effects, lack of awareness and forgetfulness, missing and discontinuing as reasons for none use. Studies have been done in Nigeria [26], Kenya [24], Senegal [16] and India [20] come up with similar findings.

### Study limitations and strengths

As strength, current study used relatively shorter reference period after delivery to identify utilization rate and it mixed both qualitative and quantitative research approaches to strengthen findings. The following limitations need to be considered when interpreting findings. Utilization rate presented was self reported by women that there may be over or under estimation. The sample sizes in some of cells are small and this may be affected statistical power. Study used 6 month reference period after delivery to identify level of iron supplement utilization; thus, still the recall bias might have been introduced.

## Conclusion and recommendations

Utilization of prenatal iron supplementation in the study area was very low (11.5%) and left far behind target that intended to increase utilization for 91 days or more days to 50% by 2016. Factors that predict under utilization among women were, maternal illiteracy, having no or less than four ANC visits, lack of knowledge of anemia and perceiving no benefit (thinking that iron has no health benefit) up on taking iron supplementation. Economically better off women had reduced utilization of prenatal iron supplementation than their counterparts. Lack of awareness towards iron supplement and perceived side effects contributed to non-use of the supplement. Forgetfulness and side effects facilitated missing to take the supplement on daily bases.

Thus, I recommend health sector in the study area to ensure early and frequent ANC visits through effective mobilization of HDAs, ensure comprehensive knowledge of anemia among pregnant women, provide comprehensive counseling on benefit, possible side effects and how to reduce them; as result, missing or stopping to take the supplement can be reduced, use HDAs to encourage and confirm pregnant women’s adherence to the supplement of iron recommended for betterment of utilization, users to adjust iron taking time with other usual events like meal time and bed time to reduce forgetfulness and education office in the study area to work hard on girls education and women empowerment as well.

## Supplementary information

**Additional file 1.** Questionnaires (English version).

## Data Availability

Datasets generated during and/or analyzed during the current study are available in corresponding Author.

## Abbreviations

ANC: Antenatal care
AOR: Adjusted odds ratio
CEB: Children ever born
COR: Crude odds ratio
DALY: Disability adjusted life years
ECSA: Ethiopian Central Statistical Agency
EDHS: Ethiopian demographic and health survey
EFDR: Federal Democratic Republic of Ethiopia
FGD: Focus group discussion
FMOH: Federal Ministry of Health
GA: Gestational age
HBM: Health belief model
HDA: Health development army
HEW: Health extension workers
HH: House hold
ID: Iron deficiency
IDA: Iron deficiency anemia
IFA: Iron folic acid
IFRR: Internal facility request and resupply
IQR: Inter quartile range
IRB: Institutional Review Board
LBW: Low birth weight
NNS: National nutrition strategy
PCA: Principal component analysis
PPS: Probability proportional to size
SD: Standard deviation
SPSS: Statistical Package for Social Science
SNNPR: Southern Nation, Nationalities and People’s Region
SRS: Systematic random sampling
WHO: World Health Organization
