# Supplementary material for: Predictors of prenatal iron folic acid supplement utilization in Wolaita, South Ethiopia: a community based cross-sectional study (quantitative and qualitative approach)
Source: BMC Pregnancy Childbirth. 2020 Apr 25;20:243. doi: 10.1186/s12884-020-02883-2 (PMC7183695; doi:10.1186/s12884-020-02883-2)
Supplement: Supplementary file 1 — Additional file 1. Questionnaires (English version). [file 12884_2020_2883_MOESM1_ESM.docx]

# Questionnaires (English version)

Questionnaire for determination of utilization of iron supplementation and associated factors (English version)

| **s. no** | | **Questions** | **Options** | | Skip to question |
| --- | --- | --- | --- | --- | --- |
|  | | Questionnaire number/code/ID | **------------------** | |  |
|  | | interviewer initials | **-----------------------** | |  |
| 1. **Socio-demographic information of respondent** | | | | |  |
| 1 | | Your age in complete years? | **-------------------------** | |  |
| 2 | | Religion | 1. Muslim 2.protestant 3.orthodox  4.other--------------------- | |  |
| 3 | | Marital status | 1. Married/living together 2. single  3. Divorced/separated 4. widowed | |  |
| 4 | | Education level | 1. Illiterate (cannot write and read)  2. Literate (cannot write and read) | | Illiterate, go to Q6 |
| 5 | | If literate, highest grade completed/attained | 1. Grade -------------------- 2. If higher education specify ----------- | |  |
| 6 | | Occupation | 1. Salaried employee  2. Farmer  3. Self employment/ business  4.house wife  5. Daily laborer  6. If other, specify--------------------------- | |  |
| 7 | | Number of children ever born including children died (CEB) | **------------------------------------** | |  |
| 8 | | How many individuals live together in your house? | **---------------------------------** | |  |
| 9 | | Estimated monthly household income in Ethiopian birr | **--------------------------** | |  |
| 10 | | Spousal Education level | 1. Literate 2. Illiterate | | If Illiterate, go to Q12 |
| 11 | | If literate, Highest grade Completed /attained | 1. Grade --------------------   If higher education specify ----------- | |  |
| 12 | | How long it takes to you to reach the nearest Health facility (HC/HP) from your house (minute/hour)? | ------------------------------------- | |  |
| 13 | | What kinds of toilet facility do the members of house hold use (observe)? | 1. Pit latrine with slab  2. Pit latrine without slab  3. No toilet facility/use bush/field/share  4. ventilated improved pit latrine(VIP)  5.other, ---------------------------- | |  |
| 14 | | What is the source of drinking water source? | 1. Pipe in the yard 2. Public stand in neighbor 3. Dug well 4. Protected spring 5. Unprotected sources | |  |
| 15 | | Does your house hold have radio? | 1. Yes 2. No | |  |
| 16 | | Does your house hold have television? | 1. Yes 2. No | |  |
| 17 | | Does your house hold have mobile telephone? | 1. Yes 2. No | |  |
| 18 | | Does your HH have bed with sponge/spring matters? | 1. Yes 2. No | |  |
| 19 | | Does your HH have electricity/solar or fanos? | 1. Yes 2. No | |  |
| 20 | | Does your HH have watch/clock? | 1. Yes 2. No | |  |
| 21 | | Does any member of your HH have saving account? | 1. Yes 2. No | |  |
| 22 | | Type of material the house made from | 1. Wood and steeliness steel sheet 2. Wood and grass 3. Concrete and steeliness steel sheet 4. Other ------------------- | |  |
| 23 | | What is the floor of house made from? | 1. Earth/mud/dung 2. Cement 3. Carpet 4. Ceramic 5. Wood 6. Others | |  |
| 24 | | What is the roof of house made from? | 1. No roof 2. Leaf/mud/grass 3. Wood/plank 4. Corrugated iron/metal 5. Concrete/cement 6. Others | |  |
| 25 | | What transport facility does the house hold own? | 1. Bicycle  2. Motorcycle  3. Mule/horse  4. Donkey cart  5. Car  6. None | |  |
| 26 | | Does the house hold own animals? | 1. Yes 2. No----------------------------- | | No, to 28 |
| 27 | | If yes, number they own  **(insert total number of each animal the house hold own)** | 1. Cows, oxen and bulls -------  2. Goats and Sheep -----------------------  3. Donkey, horse and mules ------------  4. Chickens-------------  5. others | |  |
| 28 | | Estimated area of agricultural land in local unit owned by the house hold | -------------------------------- | |  |
| 1. **Antenatal care utilization** | | | | |  |
| 29 | Have you had ANC visit during recent pregnancy? | | 1. Yes  2. No | | If no, to  Q 38 |
| 30 | If yes for Q29, where (place of ANC visit)? | | 1. Health post  2. Health center  3. Hospital  4. Other----------------------------------------- | |  |
| 31 | If yes for Q 29, how many months of the pregnancy of the name during the initiation of first visit? | | ------------------------------------------- | |  |
| 32 | If yes for Q 29, how many times have you visited the antenatal care during your recent pregnancy? | | 1. Once 2. Two times  3. Three times 4. > 4 times | |  |
| 33 | If yes for Q29, Did the health care provider talked about iron supplementation during ANC visit? | | 1. Yes 2. No | | If no, go to  Q 35 |
| 34 | If yes for Q29, what was the information that he/she provided? **(more than one answer is possible)** | | 1. Iron prevents anemia 2. All pregnant mothers need to take iron for six month of pregnancy on daily bases 3. Iron taking has little side effects like nausea, metal taste….etc. 4. Take iron before meal if there is no side effect observed 5. Take iron after meal if there is side effect 6. Others --------------------------- 7. I don’t remember | |  |
| 35 | How do you rate the quality of overall ANC counseling the health care provider gave you during your ANC visit? | | 1. very poor 2. poor 3. neutral 4. good 5. very good | |  |
| 36 | Did you think that you can face complications during pregnancy? | | 1. Yes 2. No | |  |
| 37 | During previous pregnancies did you face any life threatening conditions? | | 1. Yes 2. No | |  |
| **Knowledge regarding anemia** | | | | | |
| 38 | Have you ever heard about anemia? | | 1. Yes 2. No | | no, to 40 |
| 39 | If yes, what symptoms are explained by anemic individuals?  **(more than one answer is possible)** | | 1. Pallor  2. Tiredness  3. Lack work capacity  4. Become seriously ill  5. Loss of appetite  6. I don’t know  7. other; specify---------------------- | |  |
| 40 | If yes for Q38, What are the causes of anemia?  **(more than one answer is possible)** | | 1. Inadequate dietary iron intake  2. Not taking prenatal iron supplementation  3. Blood lose  4. Parasitic infection  5. I don’t know  6. Other; specify------------------------------ | |  |
| 41 | If yes for Q38, How is it possible to prevent anemia?  **(more than one answer is possible)** | | 1. Using iron tablet supplements  2. Proper nutrition  3. Preventing parasitic infection  4. Other; specify--------------------------- | |  |
| 42 | If yes for Q38, What are consequences of anemia you know?  **(more than one answer is possible)** | | 1. Increase risk of dying of mothers  2. Mothers become seriously ill  3. Can result in low birth weight  4. Can result in fetal death and still birth  5. Can impair growth of children  6. I don’t know  7. Others; specify---------------------- |  | |
| 43 | What symptom of anemia did you experience during previous pregnancies**?**  **ask mothers who know about anemia** | | 1. Tiredness/fatigue  2. Pallor  3. Lost appetite  4. Have been seriously ill  5. Have cold in hands and feet  6. lost concentration  7. None  8. Others---------------------------------------- |  | |
| 1. **Iron supplement utilization** | | | | | |
| 44 | Have you ever heard about iron supplementation that being given during pregnancy? | | 1. Yes 2. No |  | |
| 45 | During the recent pregnancy did anyone tell you about the importance of taking iron supplement? | | 1. Yes 2. No | If no, go to Q47 | |
| 46 | If yes who for Q41, provided the advice  (Multiple answers possible) | | 1. Health professional 2. Health extension workers 3. Community health promoters (1-5 network members) 4. Family members/Neighbors 5. Other specify: __________ |  | |
| 47 | During the recent pregnancy did you receive any message from mass media (TV, radio, newspapers) that promotes the use of iron supplements? | | 1. Yes 2. No |  | |
| 48 | Did health extension worker conduct home to home visit to your household during your recent pregnancy? | | 1. Yes  2. No | If no,  go to  Q 50 | |
| 49 | If yes for Q44, did she talk to you about iron supplementation? | | 1. Yes  2. No |  | |
| 50 | Did WDA leader conduct home to home visit to your household during your recent pregnancy? | | 1. Yes 2. No | go to Q52 | |
| 51 | If yes for Q46, did WDA discuss about issues regarding iron supplementation? | | 1. Yes  2. No |  | |
| **52** | **During recent pregnancy have you been prescribed/taken or swallowed iron supplement?** | | **1. Yes 2. No** | **If no,**  **go to 70** | |
| 53 | Have you got information on total number of days/tablets you are going to take? | | 1. Yes  2. No | If no,  go to  Q 55 | |
| 54 | If yes for Q 53, how many days/months you told totally to take? | | ------------------------------------- |  | |
| 55 | Have you received information on benefits/why you take iron tablet supplements? | | 1. Yes  2. No |  | |
| 56 | Have you received information on possible side effects of iron tablet supplements? | | 1. Yes  2. No |  | |
| 57 | How many weeks/months were you pregnant when you started taking iron supplement? | | ---------------------------- |  | |
| 58 | If started after six months of pregnancy, why not before six month of pregnancy?  **More than one answer is possible** | | 1. I had no information regarding iron 2. I started ANC Visit after six month of pregnancy 3. By the time there was no iron in the health facility 4. I felt fear that it may harm fetus 5. I felt fear that it may harm my health 6. I don’t know 7. Other------------------------------------------ |  | |
| 59 | What was the source of the iron supplement? | | 1. Health post  2. Health center  3. Hospital  4. other --------------------- |  | |
| 60 | What was the frequency that you were told to take the iron supplementation? | | 1.Take the iron supplement rarely/ some times  2. Take iron supplements on most of the days  3. Take the iron supplements on all days |  | |
| 61 | Was there any time you have got no iron tablet or received not enough/inadequate iron tablet after you went to health facility to take iron? | | 1. Yes 2. No |  | |
| 62 | **For how many days/months have you been taking the iron supplement (total number of days you have taken the tablet)?** | | **-----------------------------** |  | |
| 63 | How regularly were you taking the iron supplements? | | 1.Took iron supplement rarely/some times  2. Took iron supplements on most of the days  3. Took the iron supplements on all days/on daily bases  4. other---------------------------- |  | |
| 64 | If not on daily bases, why not on daily bases? | | 1. Due to side effects occurring after taking the supplement 2. To save insufficient or inadequate iron tablet 3. Due to forgetfulness 4. Due to advice from family member or anybody not to take on daily bases 5. I don’t know 6. Other ------------------------------------- |  | |
| 65 | Did health workers have been asking you about your adherence towards iron tablet? | | 1. Yes 2. No |  | |
| 66 | Did you stop taking iron before finishing the recommended dose? | | 1. Yes  2. No | If no, go to Q 68 | |
| 67 | If yes for Q66, why? | | 1. Due to side effects  2. Gave birth before finishing  3. Fear of multiple tablet  4. Fear of fetal weight gain.  5. Fear of health problem  6. Fear of fetal health  7. others; mention----------------------------- |  | |
| 68 | Did you experience any side effect after taking iron supplementation during your recent pregnancy? | | 1. Ye----**go to Q71**  2. No--(**thank and** **end the interview here**) |  | |
| 69 | If yes for Q68, What side effects did you experience?  **(More than one answer is possible)** | | 1. Unpleasant test  2. Nausea  3. Vomiting  4. black stool  4. Burning sensation in the stomach  5. Constipation  6. Diarrhea  7. Other, specify----------------------------- |  | |
| 70 | What were the reasons for not taking the iron supplement?  **(More than one answer is possible)** | | 1. I don’t know the importance of the supplement  2. I get enough iron from the diet  3. Fear of side effects  4. I forgot to take  5. I did not like the taste  6. Due to lack of iron at health facilities  7. Due to religious/cultural beliefs  8. I don’t know where I can get the iron tablet  9. other------------------------------------------ |  | |

| **Factors affecting utilization of iron (Based on the health belief model)** | | | | | | |
| --- | --- | --- | --- | --- | --- | --- |
|  |  | **Very disagree** | **Disagree** | **Neutral**  **Or undecided** | **Agree** | **Very agree** |
| 71 | Taking iron during pregnancy improves mothers health |  |  |  |  |  |
| 72 | Taking iron during pregnancy improves fetus/baby health |  |  |  |  |  |
| 73 | Anemia that can occur during pregnancy can have serious consequences on mother’s health |  |  |  |  |  |
| 74 | A mother who is not taking iron supplement during pregnancy can develop anemia |  |  |  |  |  |
| 75 | Anemia that can occur during pregnancy can have serious consequences on fetus/baby health |  |  |  |  |  |
| 76 | Iron tablets may cause side effects |  |  |  |  |  |
| 77 | Taking iron on daily basis is difficult |  |  |  |  |  |
| 78 | Taking iron during pregnancy may negatively affect fetal health |  |  |  |  |  |
| 79 | Taking iron supplement during pregnancy is not acceptable by family members |  |  |  |  |  |
| 80 | I can decide by myself whether to take or not to take iron during pregnancy |  |  |  |  |  |
| 81 | To use iron pregnant women needs to have comprehensive knowledge concerning iron |  |  |  |  |  |
| 82 | To improve utilization of iron by pregnant women there should be iron in health facility |  |  |  |  |  |

**Thank you!**

**Questions for qualitative component for WorHO MCH focal**

1. What ANC coverage looks like in your woreda? ----------------------------------------------
2. If its coverage is lower, what were the underlining reasons? ----------------------------------
3. How early pregnant women start ANC follow up?
4. What the utilization of prenatal iron supplementation looks like in this woreda? -----------------------------------------------------------------------------------------------------------------------
5. What could be the possible explanation for the observed coverage of prenatal iron supplementation? (Health system, client and health care provider related factors)-----------------------------------------------------------------
6. What are possible routes through which pregnant women access iron supplementation? --------------------------------------------------------------------------------------------------------------
7. Is there iron utilization status monitoring system (indicator, reporting and evaluating way to improve adherence) ----?
8. How do you describe the demand of the local women for Iron supplementation? If there is poor demand what are the underlying reasons (different factors including lack of awareness, fear of side effects, etc)?
9. What do you think about quality of counseling among health care providers regarding iron supplementation (competence, interaction with client, provision of comprehensive information)? ------------------------------------------------------------------------------------------
10. What iron supplementation distribution and its supply management looks like in the woreda? (planning the requirement, woreda stock, supplying HFs, preventing stock outs, responsibility) ------------------------------------------------------------------------------------------
11. Was there any instance in the woreda in the last year when there was no iron in the stock? If yes, how did you manage it? ----------------------------------------------------------------------
12. What cultural, religious and other misconceptions (if any) influence iron taking among pregnant women and how? ---------------------------------------------------------------------------------------------------------------------------
13. How did you try to handle those religious, cultural and other misconceptions (if any) regarding iron supplement utilization among pregnant women in the woreda? --------------------------------------------------------------------------------------------------------------------------
14. What are the challenges the woreda face to improve iron supplementation utilization among pregnant women? -------------------------------------------------------------------------------------
15. How can prenatal iron utilization among pregnant will be improved or what do you recommend to improve the utilization ------------------------------------------------------------------------------------------------------------------------------------?

***Thank you!!!***

**Questions for qualitative component for HC MCH focal or HEW**

1. What ANC coverage looks like in your catchment? ----------------------------------------------
2. If its coverage is lower, what were the underlining reasons? ----------------------------------
3. How early pregnant women start ANC follow up?
4. What are the services being given to pregnant women during ANC visit? -------------------
5. Do you provide iron tablet for women who need to be supplied? (yes, no)
6. What the utilization of prenatal iron supplementation looks like in this catchment? (coverage, availability of iron provision by health care providers) ----------------------------
7. Whom access the supplementation and when (pregnant, women at post partum period) ---
8. What maternal health condition do you use to provide iron supplementation? (anemic pregnant women, all pregnant…)?----------------------------------------------------------------
9. What are the possible routes through which pregnant women access iron tablet supplementation? --------------------------------------------------------------------------------------
10. How do you provide the iron to the beneficiaries (where you give HF or home, total tablets given per visit, counseling regarding iron)?
11. What are the possible reasons that some of pregnant women don’t take the supplementation (unwillingness, absence of supply, side effects…)? -------------------------
12. How your health facility access the iron supplementation (your stock management system)? --------------------------------------------------------------------------------------
13. Why some pregnant women stop taking the supplementation? (supply availability, provider client interaction, undesirable side effects, quality of counseling) ------------------
14. What is being done to improve adherence of iron supplementation among pregnant women who received iron? (any mechanisms to improve adherence) -------------------------
15. What do you feel about quality of counseling regarding iron supplementation utilization given for pregnant women by health care provider? ---------------------------------------------
16. Have you got any supportive supervision and follow up concerning iron supplementation (if yes, how was it)?
17. What challenges do you encounter to improve iron supplementation utilization among pregnant women? ---------------------------------------------------------------------------------
18. Do you have any recommendation to do to improve iron supplementation utilization among pregnant women? (from health professional side, system, facility, policy and strategy) -------------------------------------

**Thank you!**

**FGD guideline for pregnant women who are following ANC service**

Date of discussion: _______________

Facilitator’s name: __________________________________________

Note taker’s name: __________________________________________

Time discussion started: _______________

Time discussion ended: _______________

Region: _______________ Zone: _______________

Woreda: _______________ Kebele: _______________

Dear participants;

Good morning/afternoon. I thank you for coming here today. I am __________________ who is on the behalf of Ermias Wabeto, Abebe Sorsa and Samson G/Medhin (PhD) who are conducting a study on level and factors affecting utilization of prenatal iron supplementation in your community. Your participation in the study is on voluntary bases; however, your honest response and active participation will help us in understanding the situation better and will eventually contribute in designing appropriate interventions. All the information gathered will be kept confidential and your name will never be linked with any of the information that you will provide. Your participation is greatly appreciated.

**Background information about the discussants:**

| **No** | **Name of the participant** | **Age** | **Educational status** | **Kebele** |
| --- | --- | --- | --- | --- |
| 1 |  |  |  |  |
| 2 |  |  |  |  |
| 3 |  |  |  |  |
| 4 |  |  |  |  |
| 5 |  |  |  |  |
| 6 |  |  |  |  |
| 7 |  |  |  |  |
| 8 |  |  |  |  |
| 9 |  |  |  |  |
| 10 |  |  |  |  |
| 11 |  |  |  |  |
| 12 |  |  |  |  |
| 13 |  |  |  |  |

**Guideline for focused group discussion**

1. Should a healthy pregnant woman attend ANC? If yes, Why? If not what kind of women should get the service?
2. What do you feel about the ANC service given in your locality?

- Probe: Accessibility in terms of cost, and geography, quality of care, waiting time, attitude of health worker and confidentiality.
- Inquire: Previous experiences and impressions.
- Ask: What are the positive and negative aspects of ANC service received from health institutions
- Quality of services given (perceived quality), competence of provider, setting

1. How early or late pregnant women in your community start ANC visit?
2. What kind of services do you get during your ANC visits from the institution that attend?

- Nutritional services
- Disease prevention and control mechanisms
- Hygiene and Health promotion servies

1. Assess the knowledge of the pregnant women towards anemia.

- Ask: What do you think is the major cause of extreme fatigue and dizziness during pregnancy?
- Ask: Do you know anemia? What are its manifestations?
- Ask: what are some of its consequences in pregnancy?
- Ask: What possible prevention methods do you know?

1. Have you heard about iron tablet (show the tablet) which is given by health professionals during pregnancy?

- Ask: Why is it given for?
- Ask: How do you feel about taking a tablet during pregnancy?
- Ask: Have you ever received any education/information about taking iron tablet during pregnancy?
- Ask: Do you know any positive or negative perception or attitude in your community pertaining to it?
- Is there any religious or cultural obedience in your community not to take the tablet?
- What do you think its availability ion health facilities/health post or health centers/?

1. During this pregnancy did you take such tablet?

- Ask: Who prescribed the tablet to you?
- Ask: During the very first time IFS was prescribed to you, what kind of information did you receive from the health care provider about it?
- Ask: where the information and advices offered by the health care provider to you convincing and persuasive enough to start taking the tablet?
- Ask: Have you got information on the total number of days you are going to take the tablet?
- Ask: Have you been taking the supplement regularly on daily basis? What are the reasons for discontinuation or for not taking it regularly?

1. What should be done to improve the prenatal iron utilization among pregnant women in your community?
